# Does Higher Intraoperative Fraction of Inspired Oxygen Improve Complication Rates Following Implant-Based Breast Reconstruction?

**DOI:** 10.1093/asjof/ojac039

**Published:** 2022-05-07

**Authors:** Mallory A Rowley, Kometh Thawanyarat, Jennifer K Shah, Lawrence Cai, Elizabeth Turner, Oscar J Manrique, Brian Thornton, Rahim Nazerali

**Affiliations:** Upstate Medical University, State University of New York, Syracuse, NY, USA; AU/UGA Medical Partnership, Medical College of Georgia at Augusta University, Athens, GA, USA; Vice Provost for Undergraduate Education, Stanford University, Stanford, CA, USA; Division of Plastic & Reconstructive Surgery, Stanford University School of Medicine, Palo Alto, CA, USA; Division of Plastic & Reconstructive Surgery, Strong Memorial Hospital, University of Rochester Medical Center, Rochester, NY, USA; Division of Plastic & Reconstructive Surgery, Stanford University School of Medicine, Palo Alto, CA, USA

## Abstract

**Background:**

The surgical literature debates about whether an average intraoperative fractional inspired level of oxygen (FiO_2_) greater than 80% confers lower postsurgical complication rates. Although some evidence demonstrates minimal or no difference in short-term mortality or surgical site infections, few studies suggest negative long-term outcomes.

**Objectives:**

To the best of our knowledge, this is the first study examining the relationship between intraoperative FiO_2_ levels and postoperative outcomes in the setting of immediate prepectoral implant-based breast reconstruction.

**Methods:**

The authors retrospectively reviewed the complication profiles of 309 patients who underwent prepectoral 2-stage breast reconstruction following mastectomy between 2018 and 2021 at a single institution. Two cohorts were created based on whether intraoperative FiO_2_ was greater than 80% or less than or equal to 80%. Complication rates between the cohorts were analyzed using Chi-squared test, Fisher’s exact test, and multivariable logistic regressions. Variables examined included demographic information; smoking history; preexisting comorbidities; history of chemotherapy, radiation, or axillary lymph node dissection; and perioperative information.

**Results:**

Chi-squared and multivariable regression analysis demonstrated no significant difference between cohorts in complication rates other than reoperation. Reoperation rates were significantly increased in the FiO_2_ greater than 80% cohort (*P* = 0.018). Multivariable logistic regression also demonstrated that the use of acellular dermal matrix was significantly associated with increased postoperative complications (odds ratio 11.985; *P* = 0.034).

**Conclusions:**

Complication rates did not statistically differ in patients with varying intraoperative FiO_2_ levels outside of reoperation rates. In the setting of implant-based prepectoral breast reconstruction, hyperoxygenation likely does not lead to improved postsurgical outcomes.

**Level of Evidence: 3:**

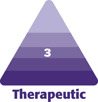

The fractional level of inspired oxygen (FiO_2_) is the concentration of oxygen in the gas mixture that is inhaled.^1^ Current literature in general surgery and anesthesiology hypothesizes that intraoperative FiO_2_ greater than 80% during endotracheal intubation confers lower postsurgical complication rates.^2,3^ In theory, increased FiO_2_ augments oxygenation during perfusion to benefit wound healing and nutrient delivery to tissues as part of the stress response in the operative setting. While the debate currently centers around whether an FiO_2_ of greater than 80% will offer no benefit, short-term benefit or, conversely, long-term complications due to hyperoxygenation, these studies have only been evaluated in the settings of abdominally based surgeries and non-thoracic surgeries.^2-4^

In the emergency setting, higher FiO_2_ contributed to lower surgical site infection (SSI) rates in colorectal surgery.^3^ Other studies that broadened the scope of outcomes concluded that increased FiO_2_ decreased SSIs in any abdominal-based surgery in the emergency setting but did not confer clinical advantages in the non-emergent setting.^2^ The nidus for our study, thus, is whether FiO_2_ offers clinical advantage in the setting of postoperative outcomes following breast reconstruction. 

In broad strokes, the literature demonstrates conflicting perspectives as studies both challenge and support the benefit of increased intraoperative oxygenation. In the context of non-emergent abdominal surgery, increased FiO_2_ did not result in lower SSIs or adverse outcomes including myocardial ischemia and atelectasis when compared with conventional FiO_2_ (near 30%).^5^ Other studies have similarly shown no significant short-term mortality advantage in the setting of non-thoracic surgeries.^6^ Recent surgical and critical literature studies demonstrate that increased FiO_2_ may both reduce and increase the risk of atelectasis, highlighting the starkly oppositional viewpoints that exist from a pulmonary perspective.^3,4,7,8^

Meta-analyses and systematic reviews additionally offer conflicting viewpoints. In a meta-analysis of 28 randomized control trials examining the role of increased vs standard intraoperative FiO_2_ on outcomes related to mortality, SSI, respiratory insufficiency, serious adverse events, and length of stay, there was no evidence to suggest a reduction in SSIs, but adverse events including mortality may increase at higher levels of intraoperative FiO_2_.^9^ An additional systematic review of 23 studies concluded that increased FiO_2_ did not decrease SSIs but may increase long-term adverse respiratory and cardiac outcomes.^1^

Our study focuses on whether intraoperative FiO_2_ is correlated with short-term postoperative complications within the first 6 months following stage 1 of reconstruction, specifically in the setting of breast reconstruction. To the best of our knowledge, this is the first study on the role of intraoperative FiO_2_ in plastic and reconstructive surgery, which may offer insight into the utility of potentially cost-effective intraoperative measures.

## METHODS

### Study Design

This study retrospectively reviewed the outcomes of female patients who underwent staged prepectoral breast reconstruction between January 2017 and October 2021 at a single institution. All reconstructions were performed by author B.T. and 3 other surgeons at this tertiary care center. The study was approved by the Stanford University IRB (IRB #49366), and charts were accessed by author E.T. All data collection was conducted in a de-identified manner. Patients were divided into 2 cohorts based on an average intraoperative FiO_2_ of greater than or equal to 80% or less than 80% using endotracheal intubation.

Demographic, comorbidity, and perioperative information collected included age, BMI, smoking history, diagnosis of diabetes mellitus or hypertension, American Society of Anesthesiologists Classification score (ASA), use of acellular dermal matrix (ADM), and mastectomy incision patterns. Age was categorized into 3 groups: less than 40 years of age, between 40 and 50 years of age, and above 50 years of age. Age categorizations were chosen in with respect to national averages of women undergoing mastectomy and reconstruction. BMI was categorized into underweight, normal, overweight, and obese groups. ASA score was categorized according to whether or not patients had an underlying severe systemic disease that warranted a physical status indicator of 3 or greater. Examples of systemic diseases that qualify for a score of 3 include severe heart disease and diabetes with vascular complications.

Charts were also reviewed for incidence of postoperative complications up to 6 months following stage 1 of reconstruction. Complications included infection, seroma, hematoma, ischemia and necrosis of the mastectomy skin flaps, nipple necrosis, explantation of the tissue expander, dehiscence, and reoperation.

### Statistical Analysis

Data were recorded in a de-identified manner and organized into tables using Microsoft Excel (Microsoft Inc., Seattle, WA). Chi-squared and Fisher’s exact tests were used to assess the variation in demographic characteristics and complication profiles between the 2 cohorts. Upon statistically significant variation between the cohorts in reoperation rates, multivariable logistic regressions were performed to calculate serially adjusted odds ratios for undergoing reoperation following the index procedure. A second multivariable logistic regression was used to evaluate predictors of experiencing at least 1 postoperative complication. Covariates included FiO_2_ greater than 80%, age, BMI, smoking history, diagnosis of diabetes mellitus or hypertension, ASA, use of ADM, and mastectomy incision patterns. *P*-values < 0.05 were considered statistically significant. All analyses were completed using Stata, version 16.1 (StataCorp, LLC, College Station, TX).

## RESULTS

Three hundred-nine female patients underwent staged prepectoral breast reconstruction between January 2017 and October 2021. Patients were divided into 2 cohorts: patients who had an intraoperative FiO_2_ of less than or equal to 80% (n = 114) and patients with breast reconstruction who had an FiO_2_ of greater than 80% (n = 195). Table 1 presents no significant difference in age, BMI, smoking history, diagnosis of diabetes or hypertension, ASA classification, use of ADM, and mastectomy incision patterns between the FiO_2_ greater than 80% cohort and FiO_2_ less than or equal to 80% using multivariate regression.

**Table 1.** Characteristics of the Study Cohort (n = 309) Among Those With FiO_2_  *<* 80 (n = 114) and Those With FiO_2_ > 80 (n = 195)

Characteristic	FiO_2_ *<* 80 (n = 114) No. (%)	FiO_2_ > 80 (n = 195) No. (%)	*P-*value
Age			
≤40 y	27 (23.7)	47 (24.1)	0.898
40-50 y	42 (36.8)	76 (39.0)	
>50 y	45 (39.5)	72 (36.9)	
BMI			
Underweight	1 (0.9)	2 (1.0)	0.999
Normal	40 (35.1)	69 (35.4)	
Overweight	41 (36.0)	69 (35.4)	
Obese	32 (28.1)	55 (28.2)	
Smoking history			
No	81 (71.1)	129 (66.2)	0.373
Yes	33 (29.0)	66 (33.9)	
Diabetes mellitus			
No	110 (96.5)	181 (92.8)	0.140
Yes	4 (3.5)	14 (7.2)	
American Society of Anesthesiologists (ASA) *>* 3			
No	80 (70.2)	122 (62.6)	0.175
Yes	34 (29.8)	73 (37.4)	
Acellular dermal matrix (ADM)			
No	0 (0.0)	5 (2.6)	0.098
Yes	114 (100.0)	190 (97.4)	
Hypertension			
No	82 (71.9)	146 (74.9)	0.570
Yes	32 (28.1)	49 (25.1)	
Mastectomy incision type			
Classic	19 (16.7)	52 (26.7)	0.069
Vertical	3 (2.6)	8 (4.1)	
NSM	86 (75.4)	118 (60.5)	
Wise	6 (5.3)	17 (8.7)	
Experienced 1+ complication(s)			
No	47 (41.2)	71 (36.4)	0.400
Yes	67 (58.8)	124 (63.6)	

Entries are frequency (percentage) unless otherwise specified. Statistical analyses conducted included Chi-squared and Fisher’s exact tests. FiO_2_, fractional level of inspired oxygen; NSM, nipple-sparing mastectomy.

Table 2 compares complication rates between the 2 cohorts. There was no significant difference between cohorts in rates of postoperative infection, seroma, hematoma, mastectomy flap ischemia and necrosis, nipple necrosis, explantation of implants, and wound dehiscence. However, rates of reoperation were significantly higher in the FiO_2_ greater than 80% group compared with the FiO_2_ less than 80% group (16.4% vs 7.0%; *P* = 0.018). In a multivariable logistic regression evaluating predictors of undergoing reoperation, including FiO_2_ greater than 80%, age, increased BMI, smoking history, diagnosis of diabetes mellitus or hypertension, ASA score, use of ADM, and mastectomy incision patterns, patients in the FiO_2_ greater than 80% cohort had significantly higher odds of reoperation (odds ratio [OR] 2.367; *P* = 0.043) (Table 3). The use of ADM was omitted from this model as no patients without ADM underwent reoperation in this dataset.

**Table 2.** Rates of Nonsurgical and Surgical Complications in the Study Cohort (n = 309) Among Those With FiO_2_ < 80 (n = 114) and Those With FiO_2_ > 80 (n = 195)

Complication	FiO_2_ *<* 80 (n = 114) No. (%)	FiO_2_ > 80 (n = 195) No. (%)	*P-*value
Infection	18 (15.8)	22 (11.3)	0.225
Seroma	47 (41.2)	93 (47.7)	0.271
Hematoma	0 (0.0)	4 (2.1)	0.157
Ischemia necrosis	16 (14.0)	44 (22.6)	0.067
Nipple necrosis	13 (11.4)	23 (11.8)	0.918
Implant explantation	14 (12.3)	30 (15.4)	0.451
Reoperation	3 (7.0)	32 (16.4)	0.018
Dehiscence	3 (2.6)	5 (2.6)	0.619

One hundred ninety-one patients experienced at least one complication due to the index procedure. Chi-squared and Fisher’s exact tests were used for statistical analysis. FiO_2_, fractional level of inspired oxygen.

**Table 3.** Patient Factors Associated With Reoperation Following the Index Procedure in the Patient Cohort (N = 309) in Basic (Adjustment for Age) and Fully Adjusted (for Age, BMI, Smoking Status, DM, HTN, ASA3, ADM, and Incision Type) Logistic Regression Models

Characteristic	Reoperation OR (95% CI)^a^	*P*-value	OR (95% CI)^b^	*P-*value
FiO_2_ level				
FiO_2_ *<* 80	1	—	1	—
FiO_2_ > 80	2.610 (1.158-5.884)	0.021	2.367 (1.027-5.454)	0.043

In total, 40 patients experienced at least one complication due to the index procedure. ^a^Adjusted for age. ^b^Adjusted for age, BMI, smoking status, DM, HTN, ASA3, ADM, and incision type. ADM, acellular dermal matrix; ASA3, American Society of Anesthesiologists (ASA) > 3; CI, confidence interval; DM, diabetes mellitus; FiO_2_, fractional level of inspired oxygen; HTN, hypertension; OR, odds ratio.

Table 4 presents a multivariable logistic regression evaluating predictors of experiencing one or more postoperative complications that included the following covariates: FiO_2_ greater than 80%, age, BMI, smoking history, diagnosis of diabetes mellitus or hypertension, ASA score, use of ADM, and mastectomy incision patterns. Only the use of ADM revealed a significant association with increased postoperative complications following stage 1 reconstruction (OR 11.985; *P* = 0.034).

**Table 4.** Patient Factors Associated With One or More Complications From the Index Procedure in the Patient Cohort (N = 309) in a Multivariable Logistic Regression

Characteristic	Average OR of 1+ complication(s) (95% CI)	*P-*value
FiO_2_ level		
FiO_2_ *<* 80	1	—
FiO_2_ > 80	1.390 (0.838-2.304)	0.202
Age		
≤40 y	1	—
40-50 y	1.401 (0.748-2.625)	0.293
>50 y	1.340 (0.683-2.628)	0.395
BMI		
Underweight	(omitted)	—
Normal Overweight	1 0.637 (0.359-1.131)	— 0.124
Obese	1.386 (0.687-2.796)	0.362
Smoking history		
No	1	–
Yes	0.758 (0.449-1.279)	0.299
Diabetes mellitus		
No	1	–
Yes	0.793 (0.269-2.359)	0.677
American Society of Anesthesiologists (ASA) *>* 3		
No	1	–
Yes	1.417 (0.808-2.483)	0.224
Acellular dermal matrix		
No	1	–
Yes	11.985 (1.207-118.983)	0.034
Hypertension		
No	1	–
Yes	1.191 (0.613-2.317)	0.606
Mastectomy incision type		
Classic	1	–
Vertical	0.589 (0.154–2.246)	0.438
NSM	1.373 (0.757–2.489)	0.297
Wise	1.321 (0.448–3.892)	0.614

One hundred ninety-one patients experienced at least one complication due to the index procedure. CI, confidence interval; FiO_2_, fractional level of inspired oxygen; NSM, nipple-sparing mastectomy; OR, odds ratio.

## DISCUSSION

SSIs and other postoperative complications are a significant cost burden in healthcare systems. Increased intraoperative FiO_2_ (above 80%) has been proposed as a cost-effective measure to potentially decrease postoperative complications but has yet to be studied within the context of breast reconstruction. The currently available general surgery and anesthesia literature is engaged in an active debate over whether increased intraoperative FiO_2_ contributes to reduced postoperative complication rates. Tissue oxygenation has been implicated in the stimulation of fibroblasts, collagen deposition, and epithelialization.^10^ It has been hypothesized that, because wound beds contain significantly less oxygen postoperatively, reduced oxygenation may contribute to impaired ability to utilize oxidative bursts to decrease bacterial load and facilitate wound healing.^9^ Hypermetabolic tissue within the wound bed has increased oxygen demand relative to supply, creating a hypoxic environment that leads to increased oxidative stress.^11^ Studies of ongoing hyperoxygenation demonstrate that increased reactive oxygen species negate the antioxidant properties of well-oxygenated tissue and result in damage at both the cellular and organ levels.^12^ By contrast, however, hypoxia notably induces transcription factors including hypoxia-inducible factor 1-alpha (HIF1-α), which promote angiogenesis and cell proliferation in hypoxic tissues.^13^ Given the varied outcomes of tissue oxygenation at the molecular level, it is conceivable that postoperative outcomes are also varied.

Increased intraoperative FiO_2_ in the setting of non-reconstructive surgical procedures has demonstrated mixed results. FiO_2_ greater than 80% only consistently contributed to lower SSIs in cases of emergent abdominal-based (eg, colorectal) surgeries.^2,3^ In the non-emergent abdominal-based surgery setting, increased FiO_2_ did not result in reduced SSIs or in increased adverse outcomes.^5^ Additional studies echo no significant clinical advantage concerning short-term mortality in non-thoracic surgeries.^4^ Conclusions regarding respiratory insufficiency secondary to atelectasis are also variable. Studies have demonstrated that increased FiO_2_ both precipitates and prevents atelectasis.^7,8^

The findings in our study demonstrate that, in the setting of prepectoral breast reconstruction, increased intraoperative FiO_2_ did not decrease postoperative rates of infection, seroma, hematoma, mastectomy flap ischemia and necrosis, nipple necrosis, implant explantation, and dehiscence, when compared with a similar cohort of patients with breast reconstruction who had an intraoperative FiO_2_ of less than or equal to 80%. Our data did, however, demonstrate increased rates of reoperation in the cohort of patients who had an intraoperative FiO_2_ of greater than 80%. While some of the aforementioned literature suggests that oxidative damage due to an average intraoperative hyperoxygenation of greater than 80% can increase the risk of postoperative complications, reoperation is unlikely to be clinically significant without additional co-existing complications such as increased rates of infection or dehiscence. This is likely an artifact in our dataset. However, a multivariate regression using FiO_2_ along with additional predictors of one or more complications demonstrated that only the use of ADM was a significant predictor of complications. There is a plethora of literature that argues for and against ADM in the setting of increased postoperative complications, particularly infection and seromas, and warrants discussion outside the framework of FiO_2_.^14-19^

While it has been hypothesized that manipulation of intraoperative FiO_2_ can be a cost-effective measure to prevent or reduce postoperative complications, our data demonstrate no evidence of clinical benefit in the setting of breast reconstruction. Our study was limited, however, to prepectoral breast reconstruction and was limited to a single-institution database that did not control for intra-institutional surgeon-specific complications. A larger sample size and further gradation of intraoperative FiO_2_ into categories beyond a binary division of greater than and less than or equal to 80% FiO_2_ may reveal more nuanced effects of FiO_2_ on postoperative outcomes. Additionally, as other studies have aptly demonstrated, separating intraoperative FiO_2_ from the effects of postoperative antibiotics can be challenging but would likely reveal additional nuanced findings if studied.

## CONCLUSIONS

Literature in the surgical and anesthesia spheres continue to debate the utility and safety of manipulating intraoperative FiO_2_ to levels above the standard (30%). To our knowledge, complication rates following reconstructive surgery have not been studied in the context of varying intraoperative FiO_2_ levels. Our data demonstrated that there was no significant difference in complication rates following prepectoral breast reconstruction in patients who had an intraoperative FiO_2_ of greater than 80% vs less than or equal to 80% except for rates of reoperation. It is unlikely that increased rates of reoperation in the FiO_2_ greater than 80% cohort are clinically relevant without other statistically significant complication differences between cohorts such as infection rates and rates of dehiscence. At this time, we believe that hyperoxygenation likely does not lead to improved postsurgical outcomes in the setting of breast reconstruction. Further study is warranted with larger sample sizes, various reconstruction procedures, and a larger gradation of intraoperative FiO_2_ levels.
